# Development of a prognostic Neutrophil Extracellular Traps related lncRNA signature for soft tissue sarcoma using machine learning

**DOI:** 10.3389/fimmu.2023.1321616

**Published:** 2024-01-09

**Authors:** Binfeng Liu, Shasha He, Chenbei Li, Zhaoqi Li, Chengyao Feng, Hua Wang, Chao Tu, Zhihong Li

**Affiliations:** ^1^Department of Orthopaedics, The Second Xiangya Hospital of Central South University, Changsha, Hunan, China; ^2^Hunan Key Laboratory of Tumor Models and Individualized Medicine of The Second Xiangya Hospital of Central South University, Changsha, Hunan, China; ^3^Department of Oncology, The Second Xiangya Hospital of Central South University, Changsha, Hunan, China; ^4^Shenzhen Research Institute of Central South University, Guangdong, China

**Keywords:** soft tissue sarcoma, neutrophil extracellular traps, lncRNA, immunotherapy, prognosis

## Abstract

**Background:**

Soft tissue sarcoma (STS) is a highly heterogeneous musculoskeletal tumor with a significant impact on human health due to its high incidence and malignancy. Long non-coding RNA (lncRNA) and Neutrophil Extracellular Traps (NETs) have crucial roles in tumors. Herein, we aimed to develop a novel NETsLnc-related signature using machine learning algorithms for clinical decision-making in STS.

**Methods:**

We applied 96 combined frameworks based on 10 different machine learning algorithms to develop a consensus signature for prognosis and therapy response prediction. Clinical characteristics, univariate and multivariate analysis, and receiver operating characteristic curve (ROC) analysis were used to evaluate the predictive performance of our models. Additionally, we explored the biological behavior, genomic patterns, and immune landscape of distinct NETsLnc groups. For patients with different NETsLnc scores, we provided information on immunotherapy responses, chemotherapy, and potential therapeutic agents to enhance the precision medicine of STS. Finally, the gene expression was validated through real-time quantitative PCR (RT-qPCR).

**Results:**

Using the weighted gene co-expression network analysis (WGCNA) algorithm, we identified NETsLncs. Subsequently, we constructed a prognostic NETsLnc signature with the highest mean c-index by combining machine learning algorithms. The NETsLnc-related features showed excellent and stable performance for survival prediction in STS. Patients in the low NETsLnc group, associated with improved prognosis, exhibited enhanced immune activity, immune infiltration, and tended toward an immunothermal phenotype with a potential immunotherapy response. Conversely, patients with a high NETsLnc score showed more frequent genomic alterations and demonstrated a better response to vincristine treatment. Furthermore, RT-qPCR confirmed abnormal expression of several signature lncRNAs in STS.

**Conclusion:**

In conclusion, the NETsLnc signature shows promise as a powerful approach for predicting the prognosis of STS. which not only deepens our understanding of STS but also opens avenues for more targeted and effective treatment strategies.

## 1 Introduction

Soft tissue sarcomas (STS) present a significant clinical challenge due to their rare occurrence, aggressive nature, and diverse subtypes derived from embryonic mesoderm (1, 2). These malignancies account for approximately 1% to 2% of all new adult cancer cases (3, 4). Given the diversity and aggressive biological behavior of STS, treating these tumors remains an immense clinical challenge (5). Although complete surgical resection combined with adjuvant or neoadjuvant radiotherapy continues to be the primary conventional treatment for localized primary STS, many patients still experience recurrence and metastasis, leading to poor prognosis (6, 7). The emergence and rapid advancement of immunotherapy have provided new insights into STS treatment (8–10). However, tumor heterogeneity limits the significant benefits of immunotherapy to only a small fraction of patients (11). Therefore, we try to addresses this challenge by introducing a novel perspective that integrates machine learning algorithms to establish a robust Neutrophil Extracellular Traps-related long non-coding RNA (NETsLnc) signature. This innovative approach not only contributes to prognosis prediction but also holds implications for durable individualized clinical management of STS.

Since soft tissue sarcoma (STS) is a highly complex disease with inter- and intra-tumor heterogeneity, an ideal biomarker should demonstrate stable performance across all tumor tissue samples. Notably, polygenic signatures have emerged as a promising approach to address this challenge, facilitated by advances in bioinformatics techniques (12, 13). However, polygenic signatures often face difficulty in clinical application due to underutilization of data information, inappropriate machine learning methods, lack of rigorous validation across different cohorts, and absence of clinical testing (11). Neutrophil Extracellular Traps (NETs) are lattice-like structures secreted by activated neutrophils, composed of Deoxyribonucleic acid (DNA) fibers, histones, and antibacterial proteins. They are responsible for entrapping and eliminating extracellular pathogens, playing a protective role in antibacterial defense (14). The process by which neutrophils secrete NETs is termed NETosis, a form of inflammatory cell death that differs from apoptosis and necrosis. Activation of neutrophils and NADPH oxidase is typically required to generate reactive oxygen species (ROS) for the formation of NETs. Increasing evidence has revealed that NETs serve multiple functions in cancer-associated inflammation (15). For example, NETs can directly or indirectly promote tumor growth, progression, and the spread of tumors to distant sites. Moreover, NETs can contribute to tumor angiogenesis and tumor-associated thrombosis (16). Concomitantly, long non-coding RNAs (lncRNAs) play a role in cancer development by affecting proliferation, migration, invasion, and chemoresistance (17). The lncRNA-related prognostic model holds potential significance in predicting the prognosis of cancer. For instance, Zihan Ding reveal that the m6A- and immune-related lncRNA signature have a robust predictive value for prognosis and immune efficacy of lung squamous cell carcinoma (18). Similarly, the NETs-related lncRNAs (NETsLnc) signature has been established and shown remarkable performance in predicting prognosis and immune infiltration in various tumors. Chen Fang et al. developed an innovative prognostic signature comprising NETsLnc that aids in predicting the prognosis of non-small-cell lung cancer (19). Nonetheless, the role of NETsLnc in predicting clinical outcomes, immunotherapy response, and the modulation of interactions between tumor cells and immune cells in soft tissue sarcomas (STS) remains largely unknown. Additionally, it is worth exploring whether the NETsLnc-based signature stands out among the numerous established STS signatures.

In this unprecedented big data era, machine learning has become increasingly important in digging rich information hidden in massive data. Multigene panels could be a potential solution to construct a roubst signature for cancer patients. Previously, Zaoqu Liu et al. successfully integrated 96 combinations of 10 commonly used algorithms into an artificial intelligence consensus program to develop a machine learning-based prognostic feature for melanoma. However, the feasibility of this integrated machine learning algorithm in constructing a prognostic model for patients with soft tissue sarcoma remains unclear (20). Therefore, we aim to identify a novel NETsLnc-related signature for the clinical decision-making of STS through this artificial intelligence consensus program consisting of 96 combinations based on ten common machine algorithms. Meanwhile, we systematically and comprehensively explored the predictive value of the NETsLnc signature in the prognosis and immunotherapy response of patients with STS, which will help to optimize the precise treatment and further improve the clinical outcomes of patients with STS.

## 2 Materials and methods

### 2.1 Public data collection and processing

**Figure 1:**
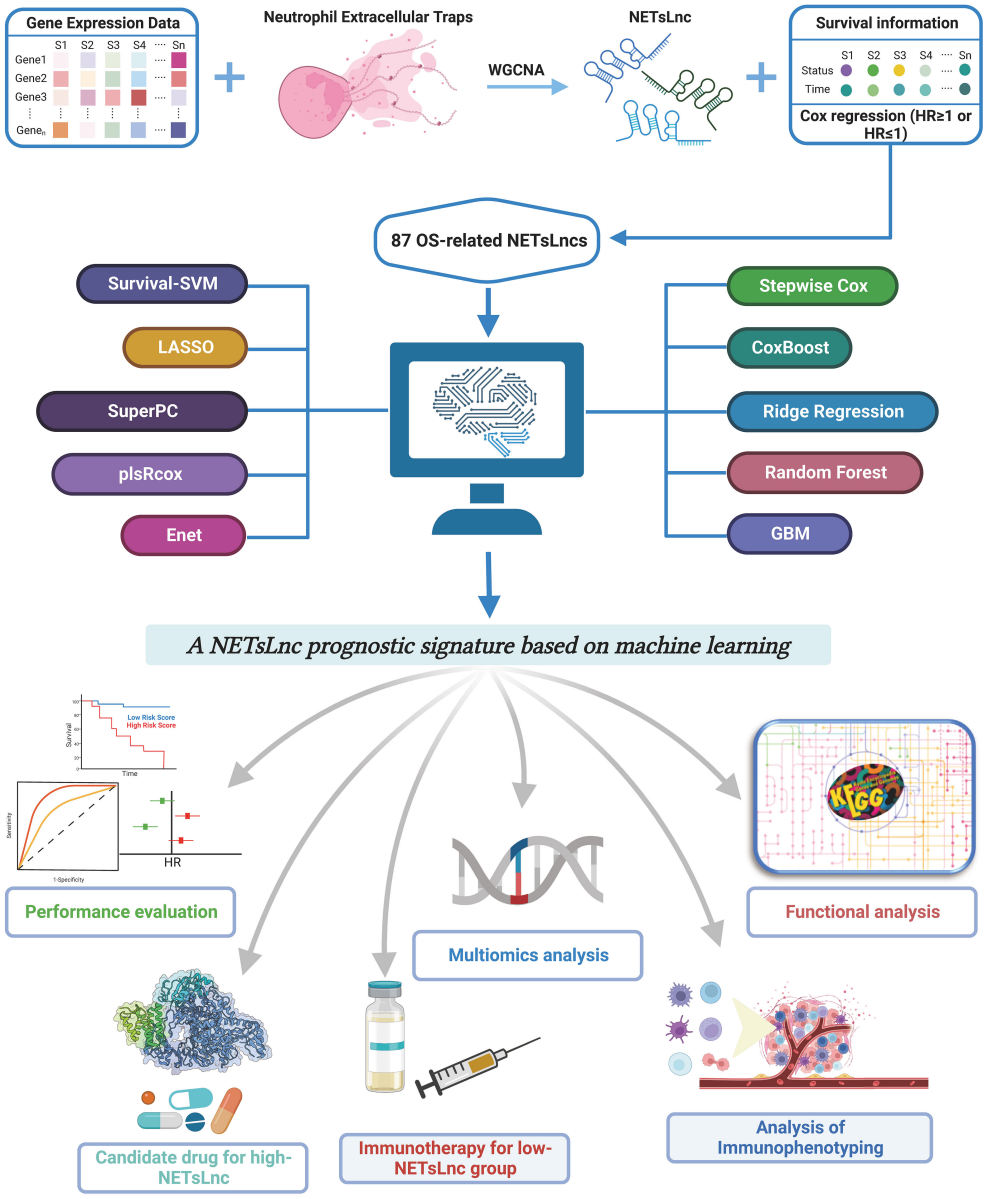
An overview of the study’s flowchart.

**Figure 1** illustrates the flowchart of the research procedure. For this study, transcriptome data, somatic mutation data, copy number variation (CNV) data, and clinical information of sarcoma patients were obtained from The Cancer Genome Atlas (TCGA, https://portal.gdc.cancer.gov/) (21), The Therapeutically Applicable Research to Generate Effective Treatments (TARGET, https://ocg.cancer.gov/programs/target) (22) and Gene Expression Omnibus (GEO, https://www.ncbi.nlm.nih.gov/geo/) database (23). After excluding samples without survival time and survival status information, a total of 969 samples were collected from four datasets: 259 samples from the TCGA dataset, 88 samples from the TARGET database, 310 samples from the GSE21050 dataset, and 312 samples from the GSE71118 dataset. The R packages “GeoTcgaData” and “AnnoProbe” were used for converting ensemble IDs to gene symbols. For RNA-seq data, normalization was performed using log2 transformation. Additionally, expression data from microarrays were normalized using the Robust Multiarray Average (RMA) method. Eligible datasets containing expression data and immunotherapy information, namely the Imvigor210 cohort and the Liu David dataset, were also screened to evaluate immunotherapy response. Finally, the NET-related genes obtained from a previously reported study were utilized for further analysis (24).

### 2.2 Identification of neutrophil extracellular traps-related long non-coding RNAs

Before identifying NETlnc, we first screen the intersected lncRNAs in all datasets. Subsequently, we utilized the R package “weighted gene co-expression network analysis (WGCNA)” was utilized to constructed a scale-free gene co-expression network based on previous research (25, 26). After ensuring that there were no missing values in the expression matrix data, the samples were clustered, and outlier samples that deviated significantly from the others were removed. Next, the gene expression matrix was transformed into an adjacency matrix to facilitate the development of an unsupervised co-expression relationship module. Furthermore, the dynamic tree-cutting method was employed to identify gene modules in the phylogenetic tree. Finally, the most correlated modules were utilized to identify significant NETlnc modules for subsequent analysis.

### 2.3 Construct NETlnc signature using machine learning-based integrative approaches.

According to previously reported research, the Z-score transformation was employed to enhance comparability among different datasets (27). Subsequently, a univariate Cox analysis based on NETlnc was conducted to identify potential survival-related genes. Next, a consensus prediction model was developed using a machine learning algorithm that combined 96 algorithms, as described in previous studies (11, 20). The most valuable signature with the highest c-index was selected through tenfold cross-validation. In this process, the STS dataset with the largest number of samples was designated as the training cohort for implementing the aforementioned algorithm, while the remaining three independent cohorts served as the test cohort, where the c-index was calculated. The model with the highest average c-index value was considered the optimal model (20).

### 2.4 Assess the prediction performance of NETlnc signature

Everyone in all cohorts was assigned a score based on the resulting model, and all datasets were stratified into low and high NETsLnc groups based on the median risk score. The “survival” package was used to draw Kaplan-Meier (K-M) survival curves to compare the differences in survival time between the different risk groups. The receiver operating characteristic (ROC) curve, C-index value, and area under the ROC curve (AUC) were used for the NETsLnc signature prediction accuracy evaluation. Meanwhile, the univariate and multivariate Cox regression analyzes were performed to confirm the independence of the novel NETlnc signature. Additionally, a nomogram was developed to better predict the prognosis of STS using the “regplot” and “rms” packages. The calibration curves and AUC values were utilized to evaluated the nomogram’s predictive value.

### 2.5 Background on the potential biological functions of NETlnc signature

To investigate the biological characteristics associated with different NETsLnc patterns, a series of analyzes were conducted. Initially, a comparison was made between the two risk groups in terms of the cancer-immunity cycles signature, which consists of 7 steps guiding frameworks for cancer immunotherapy. Additionally, the correlation between the NETsLnc score and hallmark signature, as well as the relationship between canonical immune markers and NETsLnc score, were explored. Furthermore, the correlation between these two variables was graphically depicted using a butterfly plot generated by the “corrplot” R package. Lastly, the R package “GSVA” was utilized for Gene Set Enrichment Analysis (GSEA) to identify significant functional pathways associated with different NETsLnc groups (28). The hallmark gene set and c2.cp.kegg.v7.4 were acquired from the Molecular Signatures Database (MSigDB, https://www.gsea-msigdb.org/gsea/msigdb/index.jsp).

### 2.6 The genomic variation landscape

To explore and understand molecular heterogeneity at the genomic level, we sought to map the landscape of genomic variations. As frequently mutated genes (FMGs) with high mutation frequencies are regarded as key driver genes, we initially calculated the tumor mutational burden (TMB) for each STS patient using the “maftools” package. We then identified the top 20 genes with the highest mutation frequency and compared the differences in mutation frequencies between the high and low NETsLnc groups (29). Additionally, we examined the variation in amplification and deletion burden among different NETsLnc groups based on copy number variation (CNV) data derived from GISTIC2.0 analysis. Moreover, we investigated the correlation between the NETsLnc score and the level of amplification and deletion burden based on the CNV data. In addition to TMB, we also compared the differences in the mutation impact score (MIS) and homologous recombination deficiency (HRD) between distinct NETsLnc groups to identify potential genomic signatures.

### 2.7 Delineate the immune-related characteristics of NETlnc signature

The ESTIMATE algorithm, which utilizes expression data, was employed to calculate the StromalScore, immuneScore, ESTIMATScore, and Tumor purity using the “ESTIMATE” package (30). The CIBERSORT package in the R software was utilized to assess the relative expression levels of 22 immune cells in STS samples (31). Gene sets of immune checkpoints, extracted from prior research, were used to comprehensively evaluate the immune profile of the tumor microenvironment. Additionally, differences in the Intratumor Heterogeneity, Proliferation, Leukocyte Fraction, and Lymphocyte Infiltration Signature between distinct NETsLnc groups were compared (25).

### 2.8 A comprehensive evaluation of immunotherapy

The IMvigor210 (32) and Liu David (33) datasets were employed to predict the response to immunotherapy. Initially, the NETsLnc scores were calculated in these two datasets, and subsequently, the differences in survival prognosis between patients with different NETsLnc scores were compared. Furthermore, the disparities in NETsLnc scores among patients with varying immunotherapy effects were also evaluated. Additionally, the Subnetwork Mappings in Alignment of Pathways (Submap) approach was applied to forecast the responses to anti-PD-1 and anti-CTLA-4 immunotherapy. Concurrently, the immune dysfunction and exclusion (TIDE) algorithm were implemented to predict immunotherapy responses among distinct risk groups (34, 35).

### 2.9 Screening for potential therapeutic drugs

To identify potential therapeutic agents for STS, we conducted comprehensive analyzes using the Connectivity Map (CMap, https://www.broadinstitute.org/connectivity-map-cmap) (36) and Profiling Relative Inhibition Simultaneously in Mixtures (CTRP, https://portals.broadinstitute.org/ctrp/) (37) and Profiling Relative Inhibition Simultaneously in Mixtures (PRISM, https://www.theprismlab.org/) (38). CMap is a widely-used method for searching potential therapeutic agents and targeting pathways based on gene expression profile similarities (39). The “limma” package was used to identify elevated NETsLnc subtype genes and compare them with the database features for expression similarity. The treatment sensitivity of the identified agents was then assessed through quantitative enrichment scores. The CTRP dataset contains sensitivity data for over 481 compounds, while the PRISM dataset provides sensitivity data for 1448 compounds. Both datasets include the Area Under the Curve (AUC) value of the dose-response curve, which inversely correlates with compound sensitivity (38). Our analysis focused on exploring drugs associated with the NETsLnc score and comparing the differences in AUC values between different patient groups.

### 2.10 Cell culture

The source of the soft tissue sarcoma cell line (hSS-005R, SYO-1) and human skin fibroblast cell line (HSF) was the same as described in previous studies (40). All cells were cultured in DMEM containing 10% FBS and 1% penicillin-streptomycin solution at 37°C, 95% oxygen, 5% carbon dioxide, and 95% relative humidity.

### 2.11 RNA extraction and real-time quantitative PCR (RT-qPCR)

All the procedures, like RNA extraction, reverse transcription, and RT-qPCR, were conducted as per the instructions of the kit. Firstly, the total RNA of cells was extracted using SteadyPure Quick RNA Extraction Kit (Accurate Biotechnology (Hunan) Co.,Ltd). Then, the RNA reverse transcription was performed utilizing Hifair III 1st Strand cDNA Synthesis SuperMix for RT-qPCR (Yeasen Biotechnology (Shanghai) Co.,Ltd). Finally, the Hieff qPCR SYBR Green Master Mix (High Rox Plus) (11203ES, YEASEN Biotech Co., Ltd, China) was used for RT-qPCR and the 2^^-ΔΔCT^ method was applied to calculate the relative gene expression level. **Supplementary Table 1** displays the sequences of primer used in the present study.

### 2.12 Statistical analysis

All statistical analyzes in this study were conducted using R software (version 4.0.1) and GraphPad Prism (version 9.0.0). The differential analysis for identifying differentially expressed genes was performed using the R package “limma”. The correlation between two continuous variables was assessed using Spearman and Pearson correlation analyzes. Group differences were assessed using two-tailed t-tests or one-way analysis of variance (ANOVA). Non-normally distributed variables were compared using the Wilcoxon or Kruskal-Wallis tests. ROC and calibration curves were generated using the “timeROC” and “rms” packages. A P-value less than 0.05 (both sides) was considered statistically significant.

## 3 Results

### 3.1 The role of NETs in soft tissue sarcoma

**Figure 2:**
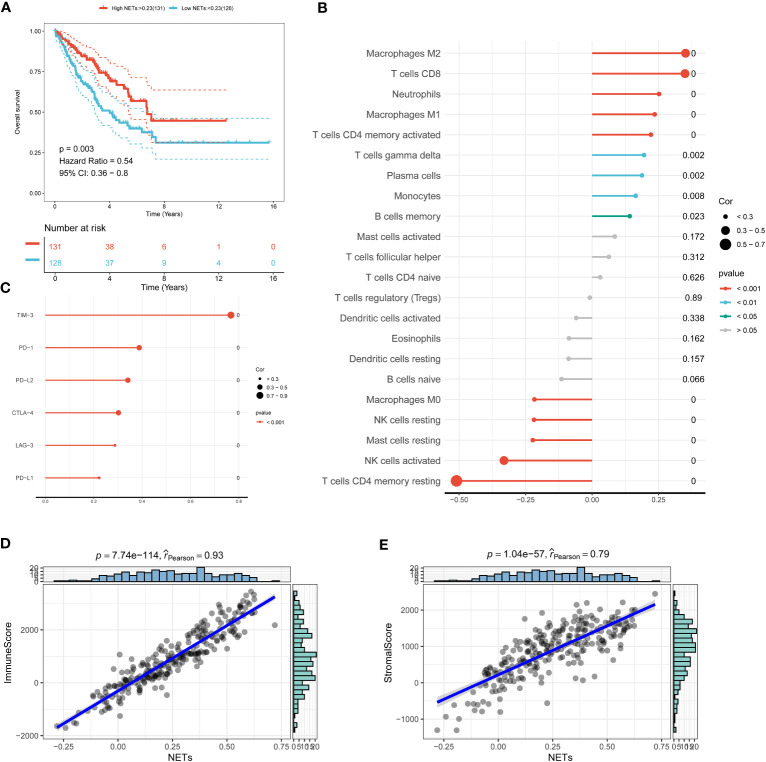
Decoding neutrophil extracellular traps (NETs) in the tumor microenvironment (TME). **(A)** The Kaplan-Meier survival curves of STS with different NETs scores. **(B)** The relationship between immune cell infiltration and NETs scores can be analyzed using Spearman’s correlation. **(C)** An association between immune checkpoint molecules and NETs score. **(D)** A correlation between immune score and NETs scores. **(E)** A correlation between stromal score and NETs scores.

The pivotal role of the tumor microenvironment (TME) in tumor initiation and progression has been underscored by a growing body of research. Therefore, it is important to investigate the immune microenvironment landscape in STS to gain new insights into the pathogenic mechanisms of this disease. In this study, we employed the ssGSEA algorithm to calculate NETs scores based on the expression of NETs-related genes, aiming to decode the crosstalk within the TME. Our results demonstrated that STS patients with higher NETs scores exhibited improved prognoses (**Figure 2A**). Additionally, we observed a significant correlation between NETs scores and immune cell infiltration, with higher NETs scores being associated with increased infiltration of immune cells such as Macrophage M2, T cell CD8, and Neutrophils (**Figure 2B**). Interestingly, a positive correlation was identified with prevalent immune checkpoints, including TIM3, PD-1, PD-L2, and CTLA4 (**Figure 2C**). Moreover, strong correlations were found between NETs scores and both stromal and immune scores, indicating a potential ability to modulate the TME (**Figures 2D, E**). Collectively, these findings highlight the close relationship between NETs and the TME, suggesting a significant impact on the tumorigenesis of STS.

### 3.2 Identification of NETsLnc

The NETsLnc signature has demonstrated remarkable predictive performance in prognosis and immune infiltration across various tumor types. In the present study, we aimed to develop a novel NETsLnc signature specific to STS by utilizing the WGCNA approach. Initially, we visualized the sample dendrogram and trait heatmap, followed by the removal of outlier samples to ensure accurate clustering (**Supplementary Figure 1A**). Then, we integrated transcriptional profiles and immune features, and using an appropriate soft threshold (β=9), we constructed a scale-free network. Subsequently, we identified 8 non-grey co-expressed modules using the dynamic shear method (**Supplementary Figure 1B, C**). Furthermore, we explored the association between these modules and NETs scores, immune scores, and matrix scores. Remarkably, the yellow and black modules demonstrated the strongest correlation with NETs, as indicated by the highest R-values (**Supplementary Figure 1D**). Consequently, the genes within these modules were designated as NETsLnc and were employed for subsequent analyzes. These findings solidify the successful identification of NETsLnc, which will serve as the foundation for constructing the prognostic signature.

### 3.3 Development of NETsLnc signature

**Figure 3:**
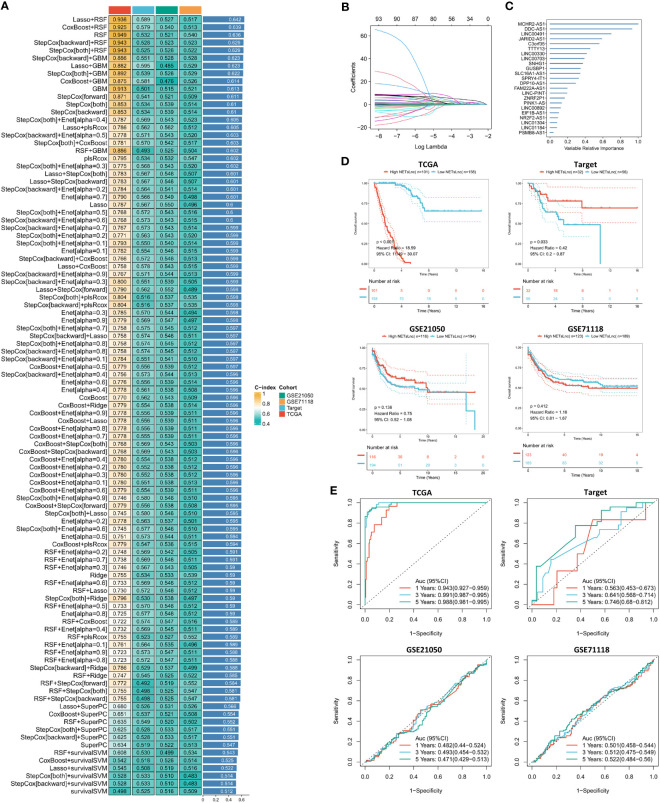
The NETsLnc signature is constructed using machine learning. **(A)** The C-index that compares 96 machine learning algorithms across four cohorts of validations. **(B)** A Lasso coefficient of each of the most useful prognostic genes. **(C)** The 23 most valuable NETsLnc. **(D)** Kaplan-Meier survival analysis between different NETsLnc groups. **(E)** A time-dependent ROC curve is presented for one year, three years and five years of OS.

Further construction of the NETsLnc signature will contribute to the prognostic assessment of STS patients. Therefore, we identified 87 genes significantly relevant to the overall survival of STS from the most relevant modules through univariate regression analysis. Subsequently, these 87 NETsLnc were incorporated into the integrated algorithm program to construct the NETsLnc signature based on machine learning. In the STS training set, we constructed prediction signatures based on the consensus of 96 algorithmic frameworks and performed ten-fold cross-validation, and computed the mean c-index of each model in all cohorts to evaluate the predictive performance of all signatures. As shown in **Figure 3A**, among the 96 signatures, the algorithm consisting of Lasso and RSF maintained the highest average c-index when constructing the final model. According to the expression data and coefficients of the model’s signature genes, we calculated everyone’s NETsLnc signature score (**Figures 3B, C**). To assess the prognostic significance of the NETsLnc model, we classified the STS individual into the high NETsLnc group and low NETsLnc group according to the median NETsLnc signature score. The Kaplan-Meier survival analysis further revealed a significantly higher risk of death in the high NETsLnc group than in the low NETsLnc group (**Figure 3D**). The same result was observed in the validation group of the TARGET cohort. However, the same results were not observed for the validation sets GSE21050 and GSE71118, which may be because these two datasets only have survival data for DFS and, thus, are less consistent with the predicted results for OS. Meanwhile, the **Figure 3E** indicates the time-dependent ROC curve of 1-year, 3-year, and 5-year OS of NETsLnc signature in TCGA (AUC: 0.943/0.991/0.988), TARGET (AUC: 0.563/0.641/0.746), GSE21050 (AUC: 0.482/0.493/0.471), and GSE71118 (AUC: 0.501/0.512/0.522).

**Figure 4:**
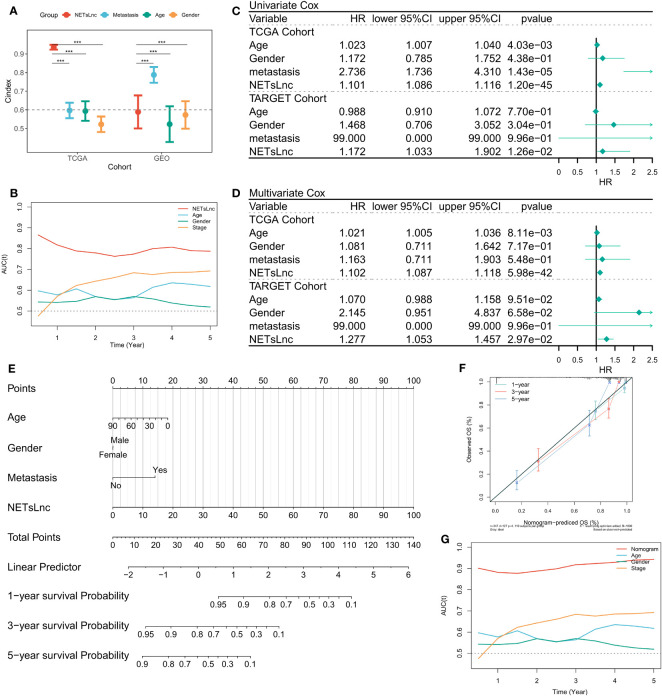
NETsLnc signature’s prognostic value. **(A)** The comparison of the prognosis prediction ability of the NETsLnc signature with the performance of common clinical features and molecular variables in the TCGA and TARGET cohorts. **(B)** The AUC of NETsLnc signature and other clinical characteristics. **(C, D)** Cox regression analysis of individual clinical variables, including the NETsLnc signature, in the TCGA and TARGET cohorts. **(E)** The nomogram is composed of the NETsLnc and clinical features to predict 1-, 3-, and 5-year survival rates. **(F)** The calibration curves of the Nomogram. **(G)** An AUC value for the Nomogram. *** < 0.001

In clinical practice, clinical characteristics such as age, gender, and metastatic status are often used for prognostic assessment and management. Therefore, we compared the predictive ability of the NETsLnc score with typical clinical characteristics in STS. The results exhibit that the NETsLnc signature was more accurate than these variables in the TCGA and GEO cohorts (**Figures 4A, B**). Moreover, the Cox regression analysis indicated the independent prognostic significance of NETsLnc signature for STS (**Figures 4C, D**). Finally, we also developed a nomogram (**Figure 4E**) based on the NETsLnc signature and clinical characteristics. The survival probability predicted by this nomogram was better consistent with the actual observation of STS (**Figures 4F, G**). Hence, these results imply that our NETsLnc marker has been successfully constructed and has strong accuracy and specificity for predicting the prognosis of STS, which will contribute to the clinical management of STS in the future.

### 3.4 The potential biological functions of NETsLnc

**Figure 5:**
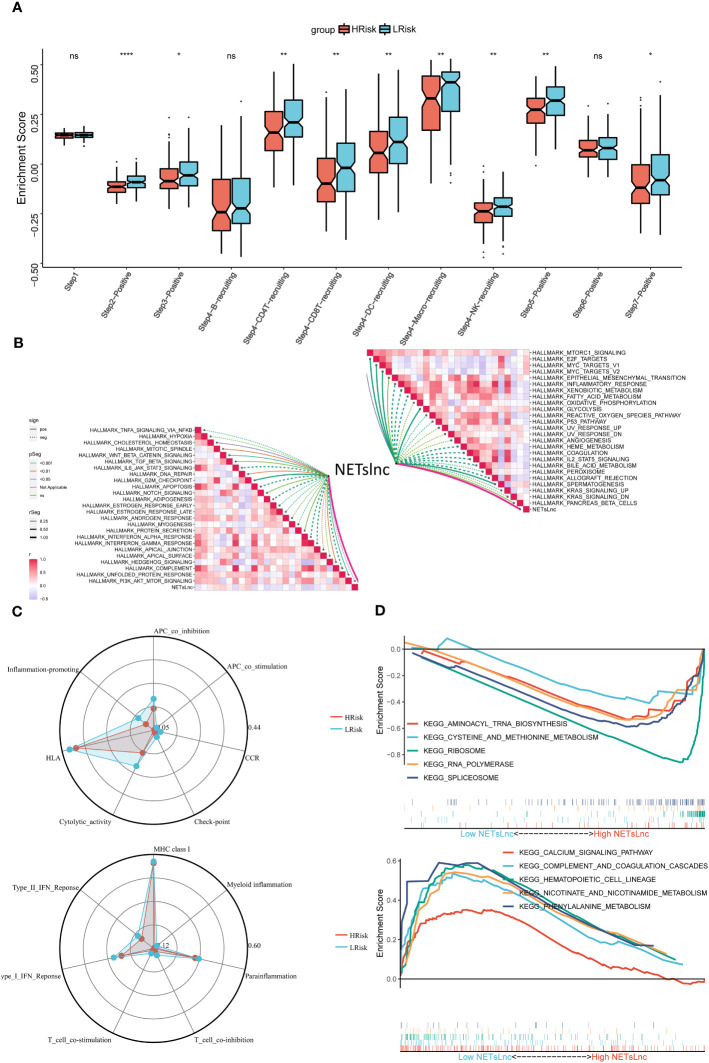
Annotation of the NETsLnc signature in the STS cohort. **(A)** Differences in cancer immune cycles between two NETsLnc signature score groups. **(B)** Correlation between NETsLnc signature scores and Hallmark terms. **(C)** Immunogram radar Chart. **(D)** The GSEA of KEGG results. * < 0.05, ** < 0.01, **** < 0.0001. ns, No significance.

Exploring the potential biological mechanisms underlying NETsLnc can provide insights into the poorer prognosis observed in STS patients with high NETsLnc scores. Our analysis of representative steps in the immune cycle revealed that the high NETsLnc group exhibited increased activity in multiple immune cycle steps (**Figure 5A**). Furthermore, NETsLnc features were found to be associated with various pathways, including myc target, Wnt beta-catenin signaling, and TGF beta signaling (**Figure 5B**). Additionally, analysis of the immune atlas radar map using the TCGA dataset showed significant upregulation of typical immune markers in the low NETsLnc group, such as cytolysis activity, inflammation promotion, and APC co-inhibition (**Figure 5C**). Through GSEA, we identified distinct enrichment patterns in the low and high NETsLnc groups. The low NETsLnc group was enriched in pathways such as aminoacyl tRNA biosynthesis, cysteine and methionine metabolism, ribosome, RNA polymerase, and spliceosome (**Figure 5D**). On the other hand, the STS cohort with high NETsLnc scores exhibited enrichment in pathways such as calcium signaling, complement and coagulation cascades, hematopoietic cell lineage, nicotinate and nicotinamide metabolism, and phenylalanine metabolism (**Figure 5D**). These findings provide novel insights into the adverse prognosis associated with high NETsLnc scores in STS patients.

### 3.5 Genomic mutation landscape

**Figure 6:**
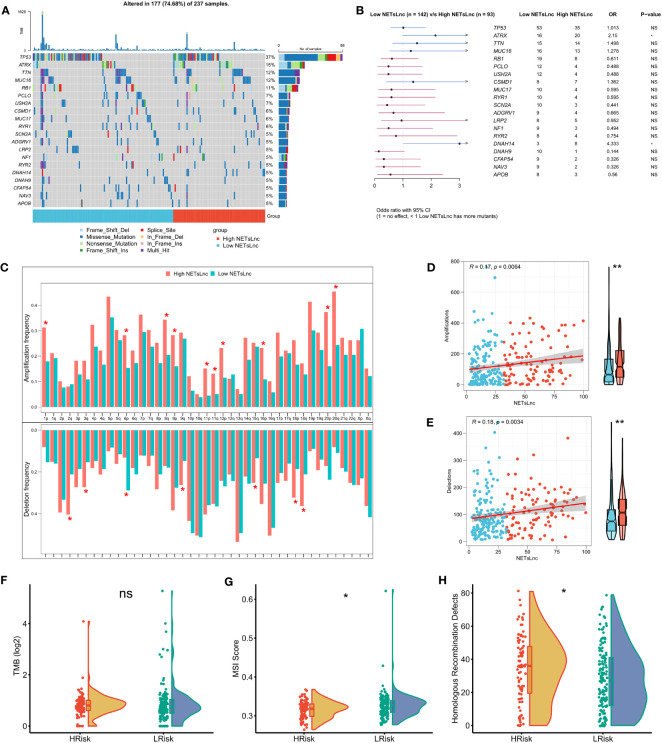
Genomic alterations associated with the NETsLnc signature in the STS cohort. **(A)** Waterfall plotting of the top 20 commonly mutated genes. **(B)** The top 20 FMGs between distinct risk groups. **(C)** The amplification and deletion frequency distributions between distinct risk groups. **(D)** Association of NETsLnc signature score with amplification frequency. **(E)** Correlation of NETsLnc signature score with deletion frequency. **(F)** The difference in TMB score between two NETsLnc signature score groups. **(G)** The difference in the MSI score between two NETsLnc signature score groups. **(H)** The difference in the Homologous Recombination Defects score between two NETsLnc signature score groups. * < 0.05.

Understanding the somatic mutation landscape is crucial for gaining in-depth insights into the disease biology, guiding treatment decisions, and enhancing the accuracy of prognosis assessment of STS. As presented in **Figure 6A**, we explored the somatic mutation landscape and visualized the top 20 FMGs in STS patients via waterfall. Notably, among these common FMGs, the mutation frequencies of ATRX and DNAH14 were significantly elevated in the high NETsLnc group than in the low NETsLnc group (**Figure 6B**). To further dissect genomic variation, we further compare and evaluate CNVs on chromosome arms between different NETsLncs groups. Strikingly, the patient in the high NETsLncs group had more pronounced deletion and amplification changes (**Figure 6C**). Meanwhile, the NETsLncs scores were positively correlated with deletion and amplification changes (**Figures 6D, E**). Although there was no difference in TMB between the high and low NETsLnc group, the patient with a high NETsLnc score exhibited an enhanced HRD burden and diminished MSI score (**Figures 6F–H**). Collectively, the low NETsLncs signature may represent a stable genomic subtype, while the high NETsLncs signature has an augmented degree of genomic instability.

### 3.6 Integrative assessment of tumor immunity microenvironment

**Figure 7:**
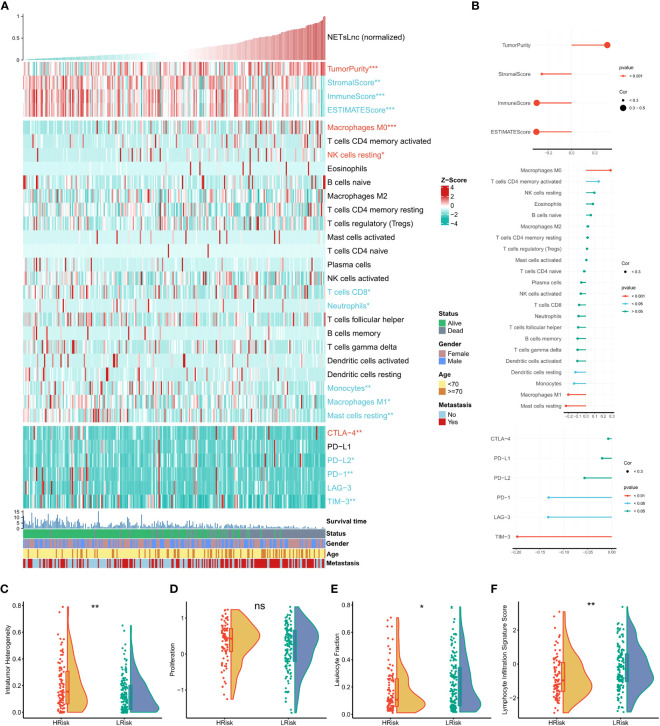
The immunity characteristics of the STS cohort relating to the NETsLnc signature. **(A)** The accsociation of the NETsLnc signature with immune score, immune infiltrating cells, and immune checkpoint. **(B)** The accsociation of NETsLnc signature and immune score, immune infiltrating cells, and immune checkpoint. **(C)** The intratumor heterogeneity level between two NETsLnc signature score groups. **(D)** The prolifferation level between two NETsLnc signature score groups. **(E)** The leukocyte fraction level between two NETsLnc signature score groups. **(F)** The lymphocyte infiltration signature score between two NETsLnc signature score groups. * < 0.05, ** < 0.01. ns, No significance.

To further explore the immune status reflected by the NETsLnc signature, we conducted a series of correlation analyzes. As presented in **Figures 7A, B**, the patient with high NETsLnc exhibited a higher tumor purity score and positively correlated NETsLnc score. In comparison, the stromal, immune, and ESTIMATE scores were lower and negatively correlated with the NETsLnc scores. Meanwhile, a high NETsLnc score was significantly positively correlated with the degree of infiltration of macrophages M0, while the degree of infiltration of macrophages M1 and Mast cells resting and the level of immunomodulators were closely related to low NETsLnc score. In addition, we display that the intratumor heterogeneity of the high-risk group was higher than that of the low-risk group. At the same time, the low NETsLnc presented a lower leukocyte fraction and lymphocyte infiltration signature score, and there was no difference in proliferation score between the two groups (**Figures 7C–F**). Hence, these results indicated that the STS patients in low NETsLnc feature is associated with a more active tumor immune response, implying their potential as candidates for immunotherapy.

### 3.7 Immunotherapy response prediction

**Figure 8:**
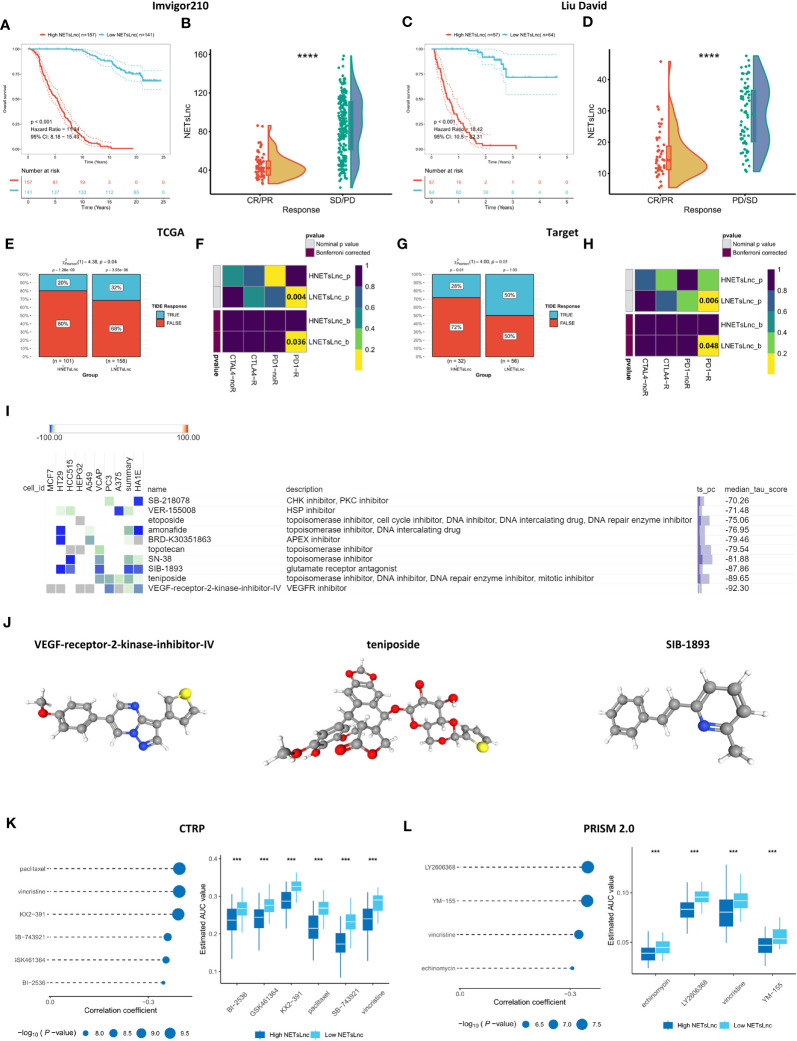
Immunotherapy response and drug sensitivity prediction. **(A)** Kaplan-Meier survival curve of OS between patients with distinct risk score in the Imvigor210 dataset. **(B)** A comparison of NETsLnc signature scores between CR/PR and SD/PD patients in the Imvigor210 dataset. **(C)** Kaplan-Meier survival curve of OS between patients with distinct risk score in the Liu David dataset. **(D)** A comparison of NETsLnc signature scores between CR/PR and SD/PD patients in the Liu David dataset. **(E)** The difference in TIDE score between the TCGA cohort with distinct NETsLnc signature score. **(F)** The submap analysis result in the TCGA cohort. **(G)** The difference in TIDE score between the TARGET cohort with distinct NETsLnc signature score. **(H)** The submap analysis result in the TARGET cohort. **(I)** Potential agents’ descriptions and their corresponding descriptions. **(J)** The three-dimensional structure of VEGF-receptor-2- inhibitor-IV, teniposide, and SIB-1893. **(K)** The potential pharmaceutical compound for STS cohort with the high NETsLnc score based on CTRP. **(L)** The potential pharmaceutical compound for STS cohort with the high NETsLnc score based on PRISM 2.0. *** < 0.001, **** < 0.0001.

The potential predictive ability of the NETsLnc signature for immunotherapy response was investigated by verifying its predictiveness in the IMvigor and Liu David immunotherapy datasets. As shown in **Figures 8A, B**, the individuals in the low NETsLnc group had significantly improved overall survival compared to those in the high NETsLnc group. Moreover, the NETsLnc scores of patients with stable disease (SD) and progressive disease (PD) were significantly higher than those of patients with complete response (CR) and partial response (PR) (**Figures 8C, D**). Consistently, the contingency table generated by TIDE and submap also supports these findings (**Figures 8E–H**). These results suggest that patients with low NETsLnc scores exhibit a higher response rate to immunotherapy and are more sensitive to PD1 inhibitors in the TCGA and Target cohorts. Collectively, these results indicate that patients with low NETsLnc scores exhibit a higher response rate to immunotherapy, which has important implications for future understanding of immunotherapy for STS.

### 3.8 Screening of potential therapeutic drugs

Screening of potential drugs offers multiple possibilities for improving outcomes for patients with STS. For individualized clinical treatment each individual, we identified potential therapeutic agents through the CMap database. Through searching for opposing expression patterns among molecular subtypes and disease phenotypes by combining the CMap database, the potential compounds and elucidating the mode of action (MoA) were identified. Ten drugs showed an individualized therapeutic potential against NETsLnc (**Figure 8I**). **Figure 8J** illustrates the 3D structures of the top three drugs, including VEGF-receptor-2-kinase-inhibitor-IV, teniposide, and SIB-1893. In addition, we also utilized data of hundreds of cell lines in the CRPT and PRISM datasets to screen potentially sensitive agents for the STS cohort with a high-NETsLnc score. The CTRP data set results revealed that the AUC for BI2536, GSK461364, KX2-391, paclitaxel, SB-74392, and vincristine was significantly lower in the high NETsLnc score group, indicating better sensitivity to these drugs (**Figure 8K**). Meanwhile, the results based on the CTRP dataset displayed that the AUC values of echinomycin, LY2606368, vincristine, and YM-155 were significantly lower in the NETsLnc high-scoring group (**Figure 8L**). Collectively, these different drugs and targeted pathways can guide individualized treatment patterns and improve clinical efficacy.

### 3.9 Verification of the NETsLnc in STS cells

**Figure 9:**
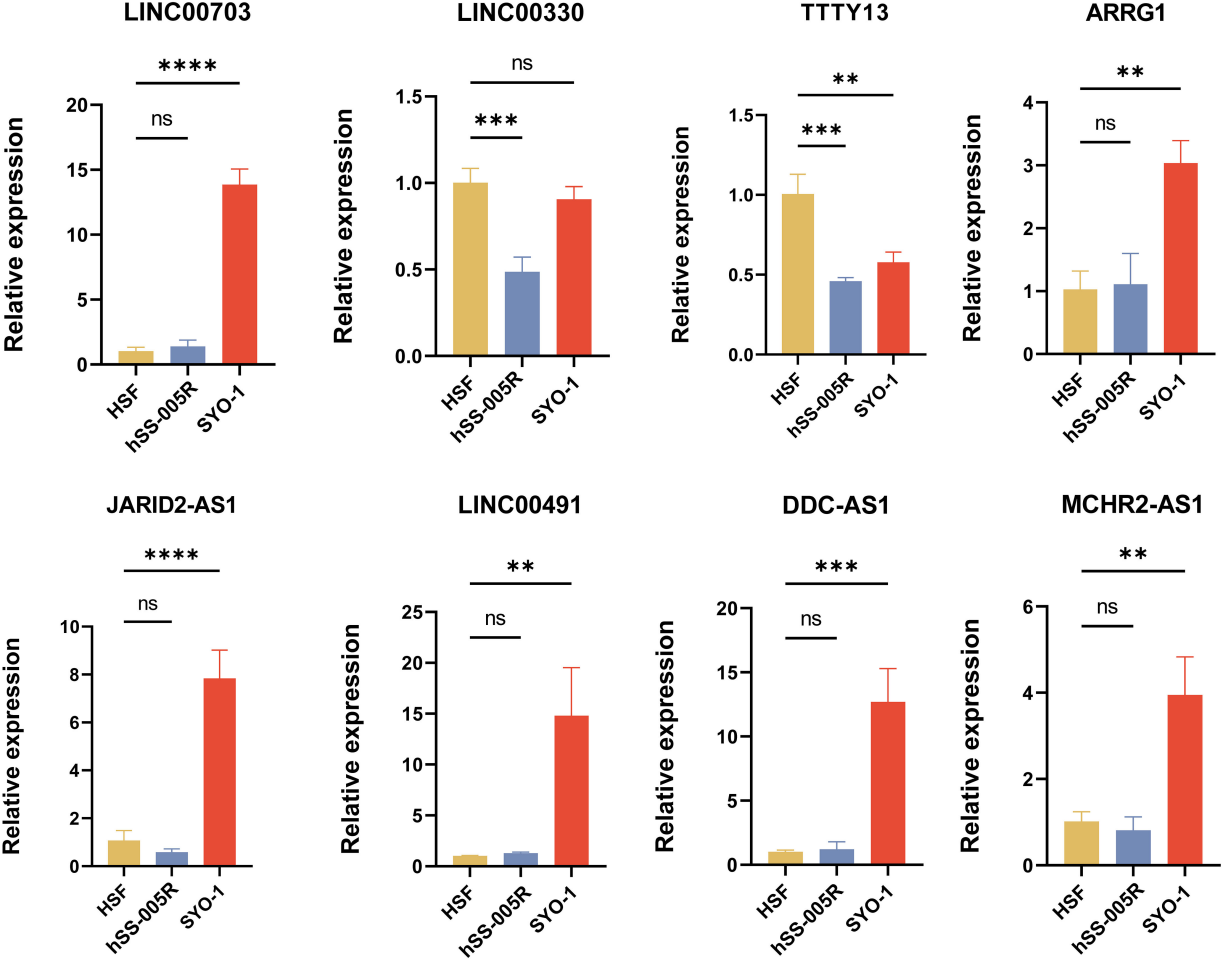
Several signature NETsLnc expressions n HSF and STS cell lines were detected by RT-qPCR. ** < 0.01, *** < 0.001, **** < 0.0001. ns, No significance.

To further confirm the effectiveness of the NETsLnc signature, we selected the top 8 characteristic NETsLnc to detect their expression in STS cells via RT-qPCR. As illustrated in **Figure 9**, there was a significant abnormal expression in these eight NETsLnc in the STS cell line. On the one hand, the LINC00703, ARRG1, JARID2-AS1 LINC00491, DDC-AS1, and MCHR2-AS1 present an improved level in SYO-1 compared to HSF, although there was no significant elevation in 005R. On the other hand, the LINC00330 was diminished in 005R, and the TTTY13 was decreased in 005R and SYO-1. Hence, these NETsLnc showed significant differences in STS cell lines, indirectly confirming the reliability and accuracy of the NETsLnc signature in STS.

## 4 Discussion

STS poses a significant clinical challenge due to its rarity, heterogeneity, and propensity for metastasis even with combined treatment strategies such as surgical resection, adjuvant radiotherapy, and chemotherapy (41). The emergence of immunotherapy has brought new hope for the treatment of tumors. Numerous clinical trials demonstrate the effectiveness of immunotherapy in various solid tumors (42). With the increasing costs of cancer treatment, there is a growing need for better-individualized assessment modalities to allocate resources more accurately to high-risk patients (43). In this context, our study aimed to bridge existing gaps by systematically investigating the relationship between NETsLnc profiles and STS prognosis, immunotherapy response, and drug efficacy using machine learning systems. This work will not only deepens our understanding of STS but also opens avenues for more targeted and effective treatment strategies.

Using the expression data of intersecting genes, we identified a consensus NETsLnc signature through an integrated pipeline comprising 10 machine-learning algorithms. Further validation on three independent datasets indicated that the model constructed by the RFS and LASSO algorithms exhibited the most outstanding performance as a predictive signature. The integration process offers several advantages: it generates a prognostic signature for STS with consistent performance, reduces the dimensionality of variables in combination with the algorithm, and simplifies and transforms the model. Additionally, a series of prognostic analyzes demonstrated that the NETsLnc score served as an unfavorable indicator and was further confirmed as an independent risk factor in the multivariate regression analysis. Furthermore, ROC and c-index analyzes revealed that the NETsLnc signature maintained high accuracy and stable performance compared to other clinical features. These results highlight the effective prognostic capabilities of the established NETsLnc signature for STS patients and its potential for significant applications in future clinical decision-making and management of STS.

Next, we investigated the underlying molecular mechanisms of NETsLnc to elucidate their association with cancer-related biological functions in different groups. We observed a positive correlation between NETsLnc signatures and several classical cancer-related pathways, which has been validated by previous studies in the context of STS. For example, in liposarcoma, leiomyosarcoma, synovial sarcoma, and fibrosarcoma, the Wnt/β-catenin signaling pathway is known to be constitutively activated, leading to enhanced proliferation and viability, with CDC25A identified as a key target gene (44, 45). Additionally, we found a significant association between high NETsLnc scores and cancer immune cycle-associated features. Research has established the importance of the cancer immune cycle as a framework for guiding cancer immunotherapy. Moreover, the immune atlas radar map demonstrated that immune features such as Cytolytic activity, Inflammation promotion, and APC co-inhibition were predominantly enriched in the low NETsLnc group, consistent with prior evidence (5, 46). Collectively, these findings suggest that the NETsLnc group exhibits notable tumor-related and immune characteristics, potentially indicating a relationship with the tumor immune microenvironment and immunotherapy.

The tumor microenvironment (TME) plays a central role in driving the initiation and progression of tumorigenesis, exerting significant influence over these critical stages in the development of cancer (47, 48). To further explore the relationship between the NETsLnc and TME, we comprehensively analyzed the immune socres and infiltration of distinct NETsLnc groups. Our study revealed that the low NETsLnc group exhibit more enhanced immune activity and several immune cells, including CD8 + T cells, Macrophage M1, and Neutrophils are more enriched. Previous studies have proven that improved CD8+ T cell infiltration may enhance immunotherapy response and prognosis (49). In the meantime, many studies have demonstrated that Macrophages can be activated with different properties in the tumor microenvironment, among which M1 macrophages have pro-inflammatory and anti-tumor effects, and an elevated M1 Macrophage distribution is associated with a better prognosis. These research are consistent with our findings, suggesting that increased infiltration and activity of immune cells may be associated with longer survival time of tumor patients. Meanwhile, this also explains to a certain extent that the patients in the NETsLnc low-risk group has a better survival prognosis.

In recent times, immunotherapy has emerged as a progressively effective alternative in tumor treatment, complementing surgical, chemotherapeutic, and radiotherapeutic approaches (50). Interestingly, we observed elevated levels of several immune checkpoint molecules, including PD-L2, PD-1, LAG-3, and TIM3, in the low NETsLnc group. These molecules showed a negative correlation with the NETsLnc score, suggesting a potential hindrance in the ability of activated immune cells to exert anti-tumor effects. However, this observation also provides an opportunity for a breakthrough in immunotherapy for STS. Supporting this, our further analysis demonstrated that patients with low NETsLnc scores exhibited better prognosis among those who received immunotherapy. Additionally, the population with good immunotherapy response had lower NETsLnc scores, highlighting the potential benefits of immunotherapy for the low NETsLnc group. Building upon this hypothesis, it is predicted that the low NETsLnc group may exhibit a favorable response to anti-PD-1 drugs compared to the high NETsLnc group. Consequently, this signature may open up new avenues for the development of more targeted and effective immunotherapeutic strategies for STS.

To date, chemical drug-mediated chemotherapy is an indispensable strategy for tumors (51). We mine representative potential therapeutics using the Camp dataset and then display their 3D structures. Additionally, we analyzed the drug susceptibility data of multiple databases to further explore potential drugs suitable for the high NETsLnc group for prognosis improvement. By analyzing the data in CTRP, we observe that the high NETsLnc group has more sensitivity to BI2536, GSK461364, KX2-391, paclitaxel, SB-74392, and vincristine. Meanwhile, the results based on the CTRP dataset displayed that the individual in the NETsLnc score group was sensitive to echinomycin, LY2606368, vincristine, and YM-155. Interestingly, both results demonstrated that vincristine might have a better therapeutic effect on STS with high NETsLnc scores. Vincristine, as a first-line antineoplastic drug, plays an integral role in the chemotherapy of patients with soft tissue sarcoma (52). Given the above, these potential therapeutic drugs may provide novel hope for STS treatment and achieve clinical precision treatment.

Moreover, we detected the expression of several signature NETsLncs in STS cell lines. Some NETsLncs have been reported to play important roles in the progression of different cancers. LINC00491 is involved in the progression of multiple cancers through miRNA sponges (53–55). For instance, Wei Wang et al. reported that LINC00491 was overexpressed in liver cancer and can promote live cancer cell growth and metastasis by sponging miR-324-5p/ROCK1 (53). LINC00703 may be a tumor suppressor in gastric and non-small cell lung cancer (56, 57). However, the significance of these NETsLnc in the development of STS is still lacking.

Although we have included as many prognostic and treatment cohorts as possible for rigorous bioinformatics analysis, further validation of these findings in a larger cohort or across different subtypes of STS would provide more robust evidence supporting the utility of this novel signature. While our study has promising strengths, it is crucial to acknowledge certain limitations. initially, the patients included in this study were from retrospective cohorts, and the prognostic signature constructed needs validation in prospective cohorts to ensure its predictive feasibility. Secondly, the roles and mechanisms of the relevant signature NETslnc in STS require further *in vitro* and *in vivo* experimental exploration in future studies. Thirdly, the impact of the signature as revealed in the study for drug selection necessitates more rigorous exploration in corresponding drug treatment cohorts.

## Conclusion

Herein, we constructed a robust consensus NETsLnc signature based on multiple machine learning algorithms, which is beneficial for the prognosis and immunotherapy response prediction of STS patients. This work not only deepens our understanding of STS but also opens avenues for more targeted and effective treatment strategies.

## Data availability statement

The original contributions presented in the study are included in the article/**Supplementary Material**, further inquiries can be directed to the corresponding author/s.

## Author contributions

BL: Validation, Writing – original draft. SH: Writing – original draft. CL: Data curation, Writing – review & editing. ZQL: Data curation, Writing – review & editing. CF: Methodology, Writing – review & editing. HW: Data curation, Writing – review & editing. CT: Funding acquisition, Writing – review & editing. ZHL: Funding acquisition, Writing – review & editing.
