# Supplementary figures and images for: Development of a prognostic Neutrophil Extracellular Traps related lncRNA signature for soft tissue sarcoma using machine learning
Source: Front Immunol. 2024 Jan 9;14:1321616. doi: 10.3389/fimmu.2023.1321616 (PMC10803471; doi:10.3389/fimmu.2023.1321616)

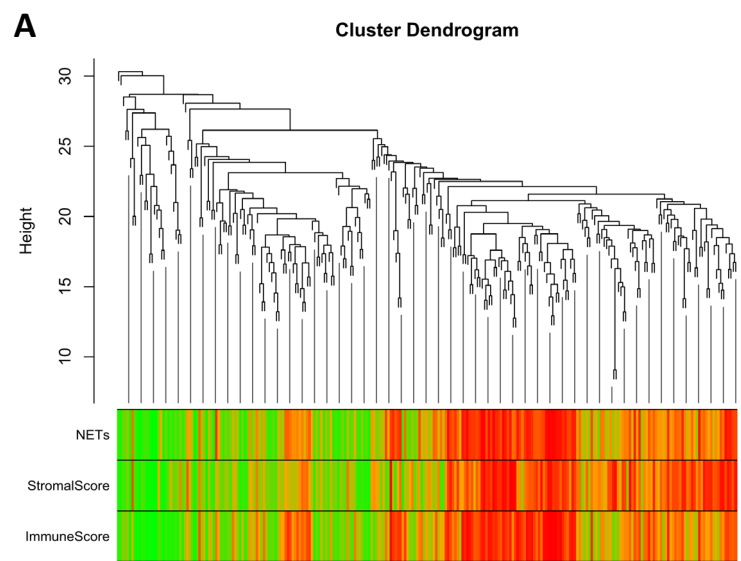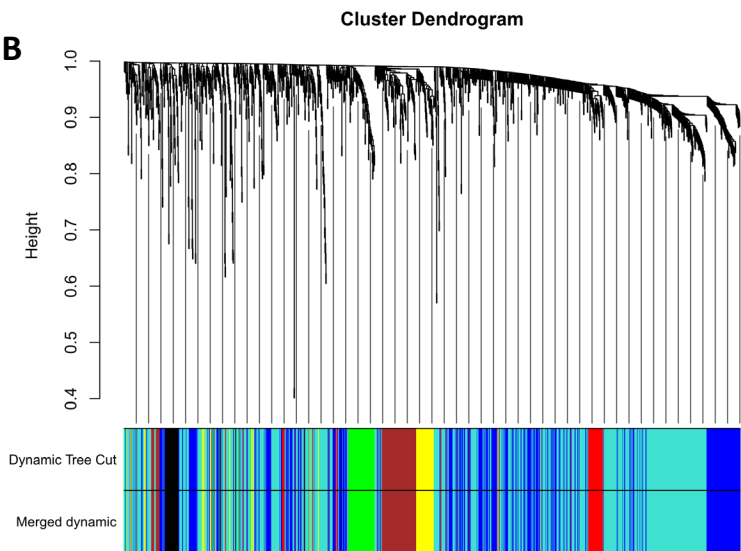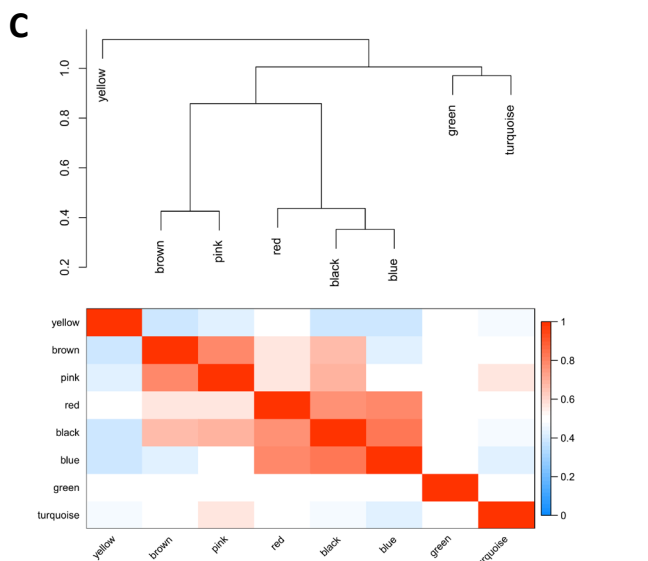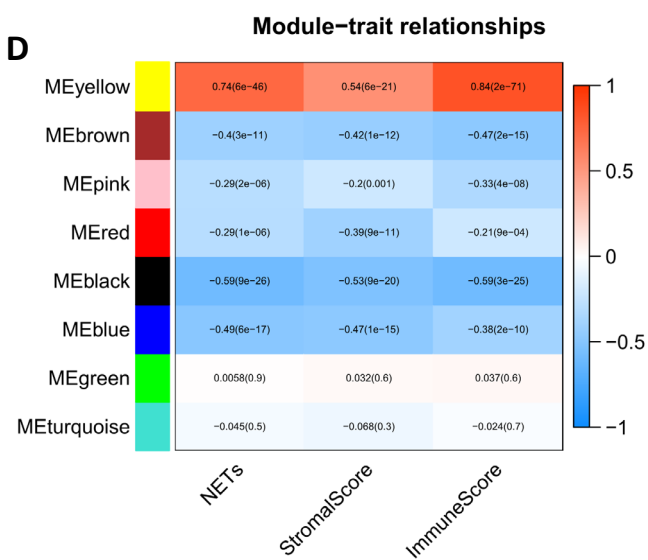

Supplement: Supplementary file 1 [file DataSheet_1.pdf]
