# Supplementary material for: Development of a prognostic Neutrophil Extracellular Traps related lncRNA signature for soft tissue sarcoma using machine learning
Source: Front Immunol. 2024 Jan 9;14:1321616. doi: 10.3389/fimmu.2023.1321616 (PMC10803471; doi:10.3389/fimmu.2023.1321616)
Supplement: Supplementary file 2 [file Table_1.docx]

| Gene | Primer Sequence (5'-3') |
| --- | --- |
| GAPDH (F) | CAGGAGGCATTGCTGATGAT |
| GAPDH (R) | GAAGGCTGGGGCTCATTT |
| LINC00703 (F) | TGGTTGCTTCCTGCCCTTGAG |
| LINC00703 (R) | CCGACGGTGCAGCCTTCAG |
| LINC00330 (F) | CATTGGAACGGCTGCATCTGTG |
| LINC00330 (R) | TTCCTCTTGTCGTGTGCTCTGG |
| TTTY13 (F) | GCTTGGCTGGGACCTGAATTTG |
| TTTY13 (R) | CAGGAGAGGGCAGACATGAGC |
| APRG1 (F) | TGCCTGTGCTTGCTGTGAGTC |
| APRG1 (R) | ATGTGCTGCCCTTGTCCACTAG |
| JARID2-AS1 (F) | TCCACGCTCCAAGACAAGGTTG |
| JARID2-AS1 (R) | GCATTGCAGAGGGCTGGGTAG |
| LINC00491 (F) | CCAGTCTTCTGCGTCGCTCAC |
| LINC00491 (R) | CACGGGAGGAGACAAGATGGC |
| DDC-AS1 (F) | CACAAGTCCACCTGGCTCCAAG |
| DDC-AS1 (R) | GGCTGCATCGGCTTCTCCTG |
| MCHR2-AS1 (F) | AGGAGTGCGAGGGTCTCTGAG |
| MCHR2-AS1 (R) | TCGGGCGTTTGGCTGTTCC |

**Table S1: The primer sequences for qRT-PCR.**
